# Patients’ priorities and expectations on an EU registry for rare bone and mineral conditions

**DOI:** 10.1186/s13023-021-02069-9

**Published:** 2021-11-03

**Authors:** Muhammad Kassim Javaid, Marina Mordenti, Manila Boarini, Luca Sangiorgi, ERN BOND Working Group, Ingunn Westerheim, Inês Alves, Rebecca Tvedt Skarberg, Natasha M. Appelman-Dijkstra, Corinna Grasemann

**Affiliations:** 1 Nuffield Department of Orthopaedics, Rheumatology and Musculoskeletal Sciences, University of Oxford, Oxford, OX3 7LD UK; 2 Department of Rare Skeletal Disorders, IRCCS Istituto Ortopedico Rizzoli, Bologna, Italy; 3 Osteogenesis Imperfecta Federation Europe (OIFE), Eindhoven, The Netherlands; 4 Associação Nacional de Displasias Ósseas (ANDO), Evora, Portugal; 5 Department of Internal Medicine, Division of Endocrinology, Leiden University Medical Centre, Leiden, Netherlands; 6 Department of Pediatrics, Division of Rare Diseases, Ruhr-University Bochum, Bochum, Germany

**Keywords:** Rare bone and mineral conditions, Rare disease registries, Osteogenesis imperfecta, Survey, Natural History

## Abstract

**Background:**

Understanding the natural history of rare bone and mineral conditions is essential for improving clinical practice and the development of new diagnostics and therapeutics. Recruitment and long-term participation in registries are key challenges for researchers.

**Methods:**

To understand the user needs, the European Reference Network on Rare Bone Diseases (ERN BOND) and European Patient Advocacy Groups developed and implemented a multinational survey about the patient’s preferred database content and functionality through an iterative consensus process. The survey was disseminated by national and international patient groups and healthcare professionals. The findings were analysed using descriptive statistics and multivariate regression.

**Results:**

There were 493 eligible responses from 378 adults, 15 children and 100 parents, guardians or carers (PGC) across 22 rare bone and mineral conditions. Osteogenesis imperfecta constituted 53.4% of responses. Contents related to improving treatment and medical services scored the highest and contents about anxiety and socializing scored less highly. Additional content was recommended by 205 respondents. Respondents preferred data entry by their Healthcare Provider (HCP). However, less than 50% of adults received followup from their specialist HCP at least annually and 29% were followed up as needed.

**Conclusions:**

This survey of individuals, their family, guardians and carers has prioritised the key components for an EU-based rare bone and mineral condition research database. The survey highlights issues around collecting psychosocial impacts as well as measures of HCP trust. The survey demonstrated that using only specialist centre visits for data collection, while preferred by patients, will miss a substantial number of individuals, limiting generalisability. Combined HCP and patient platforms will be required to collect representative and complete natural history data for this patient group.

**Supplementary Information:**

The online version contains supplementary material available at 10.1186/s13023-021-02069-9.

## Background

Understanding the natural history of diseases in terms of the type and severity of complications, progression, impact on quality of life and other clinical endpoints is essential for improving patient care pathways and development of new diagnostics and therapeutics [1]. While there are multiple guidelines for rare disease registries [2–9], including the FAIR principles [10], specific challenges for natural history studies in rare diseases remain unaddressed. These include recruiting enough participants for representative results, variability in diagnostic accuracy and ensuring sufficient long-term engagement of patients to be able to describe the natural history of events over the lifespan. Poor recruitment and short-term engagement are most likely due to the lack of addressing participant’s wishes and desires. While engagement with patients and patient groups are recommended in a number of guidelines [6, 11], there are few data describing these needs of people with rare bone and mineral conditions.

European Reference Networks (ERNs) are virtual networks involving expert Healthcare Providers (HCPs) in rare diseases across Europe [12]. ERN BOND is the European Reference Network on Rare Bone Diseases and includes 38 highly specialized HCPs from 10 EU Member States and 18 affiliated centers from 11 EU Member States in combination with designated European Patient Advocacy Groups (ePAGs). Within the structure of ERN BOND, Working Group 5 (WG5) aims to develop the specification for an EU-based registry to advance the understanding of the natural history of rare bone and mineral conditions. We here present the findings from a survey of patients, parents, families, guardians and carers for their views on the requirements of such a registry. The aim of the study was to describe the preferences of patients for an EU-based registry to describe the natural history of individuals with a rare bone disease.

## Methods

The study was a cross sectional study and conducted online in multiple languages. The survey items were co-produced by patient representatives from ePAGs, and WG5 from ERN BOND using an iterative consensus framework.

The survey was first piloted in English and then translated from English to eight respective BOND languages by the eTranslation tool provided by EU Commission and reviewed by native speakers (Czech-ZS, Dutch-NAD, Estonian-KM, German-CG, Italian-MM/MBo, Portuguese-IA, Swedish-EA) (see Additional file 1 for English version of the survey). The same tool was used to translate the answers given to the open-ended queries from each native language into English.

The survey consisted of 35 items on: (i) terms and conditions (1 item); (ii) general information (3 items); (iii) content of the research database (19 items rated on Likert scale (0 = not interested at all to 10 = very interested)); (iv) aspects of data entry (9 items); (v) survey feedback, 2 open-ended questions. We used the SurveyMonkey® platform for the survey. The survey was disseminated using multiple channels: through Healthcare Providers within BOND, national and international patient groups as well as through the BOND website (http://ernbond.eu/). The survey ran from July 1st until October 1st 2019 with the non-English surveys available from August 8th–22th (depending on language) until September 30th 2019. In the pilot phase the responses were checked and questions items adapted as needed. Since this was a non-interventional survey, no ethical approval was required although consent for the use of the data derived was requested.

### Data analysis and statistical evaluation

A total of 939 individuals participated to the survey. From this population, the following respondents were excluded: (i) respondents who did not have a rare bone and mineral condition, as defined by a prevalence of less than 1:2000 [13] affecting primarily the skeleton; (ii) respondents who refused the terms and conditions; (iii) and those who did not answer any of the items related to the database content. As the questions were not modified between the pilot and main survey, the responses were combined. The data were stratified according to group of respondent (adult, child (0–15 years) and parent/guardian or carer (PGC)) and summarized using descriptive statistics, with percentages calculated based on the total number of patients who answered each question. Results were described using histograms, box and whisker plots with median, inter-quartile range and outliers. The rating for the content items was simplified by grouping together the preference values 8, 9 and 10 to reflect high interest and 0–3 to reflect low interest. Ordinal logistic regression methods were used to compare ordered outcomes by type of respondent using STATA/IC vs 15.1 (StataCorp, TX, USA). Statistical significance was set at a *p* ≤ 0.05.

Given the small number of responses from children, those were not considered in the quantitative analyses, but were included in text analyses. As many responses came from adults with Osteogenesis Imperfecta (OI) due to the very successful dissemination via the patient representative groups, we compared responses between individuals with OI and those with other rare bone conditions.

A content analysis of the two open-ended questions was performed. Two researchers conducted a systematic examination of the text to detect the meaning units labelled with a code; thereafter the list of codes with similar meaning was collapsed in categories, aligned with the queries’ core theme: major topics (Q.34) and additional suggestions (Q.35). The results of the content analysis were presented according to the main theme as defined by respondents’ feedback. Results were summarized with descriptive statistics, using patient and quote number and percentage in each group of respondent. Comments were analyzed using ATLAS.ti software (version 8.4.24 Windows, ATLAS.ti Scientific Software Development GmbH).

## Results

**Fig. 1:**
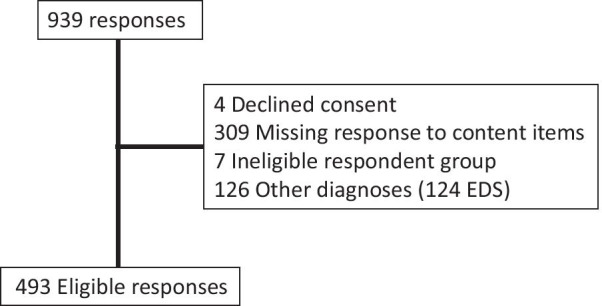
Survey responses

The English version of the survey has been added in the Additional file 1. After excluding responses from ineligible patients (Fig. 1), a total of 493 responses were collected from 378 adults, 15 children and 100 PGCs. Responses from 446 patients were excluded for the following reasons: 4 declined consent, 309 had missing content, 7 belonged to an ineligible respondent group and 126 had an excluding diagnosis (mostly Ehlers Danlos syndrome spectrum). Missing content accounted for 33.1% of all consented responses and occurred where participants completed their diagnosis but did not complete any of the preference questions, which as the primary objective for the survey. The proportion with missing content was higher for specific languages (French (39.3%), Italian (37.5%) and English (34.5%) (*p* < 0.001); children with rare diseases (37.7%, *p* = 0.019) and diagnoses ( ‘Don’t know’ (n = 12, 48%), MED (n = 1, 50%), Ollier/ Mafucci (n = 3, 50%) *p* < 0.001). Of the 493 eligible response, the response counts by language and disease type are shown in Table 1 and Table 2. English was the most common survey language used, possibly due to the longer duration this survey was open compared with other languages. It is likely that respondents to the English version included European individuals outside the UK.

**Table 1:** Completion of patient survey by language

Language	Number of responses	Percentage
English	184	37.3%
French	103	20.9%
Dutch	56	11.4%
German	55	11.2%
Portuguese	43	8.7%
Italian	35	7.1%
Swedish	10	2.0%
Estonian	7	1.4%
Total	493	100

**Table 2:** Rare bone and Mineral condition type of respondent

Condition	Adult with rare disease	Child with rare disease	Parent/guardian/carer	Total
	(n)	(n)	(n)	(n)
Achondroplasia	3	0	8	11
Aggrecan-related bone disorder	0	0	1	1
Arthrogryposis Multiplex Congenita	0	0	1	1
Diastrophic Dysplasia	1	0	0	1
FD/MAS	19	0	3	22
Fibrodysplasia Ossificans	12	2	6	20
Hereditary Multiple Exostosis	14	0	4	18
Hypoparathyroidism	4	0	0	4
Hypophosphatasia	18	0	2	20
Klippel Feil syndrome	1	0	0	1
Multiple Epiphyseal Dysplasia	0	0	1	1
Nail-Patella Syndrome	0	1	0	1
Ollier disease / Maffucci syndrome	2	0	1	3
Osteogenesis Imperfecta—Other Type	17	1	9	27
Osteogenesis Imperfecta—Type I	86	3	26	115
Osteogenesis Imperfecta—Type III	47	1	10	58
Osteogenesis Imperfecta—Type IV	20	2	5	27
Osteogenesis Imperfecta—Unknown Type	27	2	7	36
Osteopetrosis	2	0	0	2
Pseudoachondroplasia	0	0	1	1
Pseudohypoparathyroidsim	0	1	0	1
SAPHO	43	0	3	46
Sotos Syndrome	0	0	1	1
Spondylo-Epiphyseal Dysplasia	2	0	2	4
Stickler syndrome	0	0	1	1
XLH	51	2	4	57
Don't Know	9	0	4	13
Total	378	15	100	493

Of the disease groups, Osteogenesis imperfecta was the highest frequency rare disease 263/493 (53.4%). Overall, 21.3% of all respondents were already participating in a registry: 21.7% of adults and 20% of PGCs. When asked about the preferred and most descriptive name for the registry, 313 respondents gave one choice, while 128 respondents gave two to four choices. Research database was the most popular choice (65.6% of adults and 52% of PGCs).

The rating of the importance of each of the *proposed content items* by the respondents is shown in Table 3. All of the proposed database features were valued as interesting by most respondents, scoring a median of least 6 out of 10. Most of the proposed features were highly rated by over 50% of the respondents across the two groups. Contents related to improving treatment and healthcare services scored highest whereas contents related to anxiety & self-confidence (“To be able to share that I have anxiety and self-confidence problems”) and socialising (“To be able to share my experience in making friends, socialising and having relationships”) had the lowest ratings. When the preference for individual content was compared by type of respondent (adult vs. PGC), few differences were found. There was a wide spread of interest scores within each type of respondent, reflecting clear individual differences (Additional file 2: Fig. S1).

**Table 3:** Reported interest for proposed research database features: high interest (8–10/10) vs Low interest (1–3/10)

Proposed database feature		Adult	Parent/guardian or carer	*p* value
To help educate and increase the knowledge of doctors and health care professionals	High	346 (91.5%)	94 (94.0%)	NS
	Low	4 (1.1%)	1 (1.0%)	NS
To help find better treatments	High	343 (90.7%)	96 (96.0%)	NS
	Low			NS
To help provide better services and support for patients	High	334 (88.4%)	92 (92.0%)	NS
	Low			NS
To allow researchers across the world access to my unidentified information (anonymised) for research approved by the Rare Bone Diseases European Reference Network, BOND	High	302 (79.9%)	88 (88.0%)	NS
	Low			NS
To be able to share medical information about my disease with other medical staff in an emergency	High	297 (78.6%)	87 (87.0%)	NS
	Low	16 (4.2%)	6 (6.0%)	NS
To be able to describe how the disease affects me (e.g., pain and tiredness)	High	295 (78.0%)	79 (79.0%)	NS
	Low	15 (4.0%)	2 (2.0%)	NS
To help find ways to get an earlier diagnosis	High	289 (76.5%)	89 (89.0%)	*p* = 0.006
	Low	19 (5.0%)	1 (1.0%)	NS
To be able to share medical information about my disease with my doctors	High	278 (73.5%)	82 (82.0%)	NS
	Low	20 (5.3%)	5 (5.0%)	NS
To be able to share my experience in getting correctly diagnosed	High	256 (67.7%)	79 (79.0%)	*p* = 0.03
	Low	29 (7.7%)	3 (3.0%)	NS
To have the possibility to be contacted by other researchers that have expertise of my disease about new studies I may want to get involved with	High	250 (66.1%)	78 (78.0%)	*p* = 0.02
	Low	21 (5.6%)	6 (6.0%)	NS
To be able to share the impact of the disease on studying and/or working	High	244 (64.6%)	71 (71.0%)	NS
	Low	21 (5.6%)	6 (6.0%)	NS
To allow drug companies across the world access to my unidentified information (anonymised) for research approved by the Rare Bone Diseases European Reference Network, BOND	High	243 (64.3%)	72 (72.0%)	NS
	Low	27 (7.1%)	8 (8.0%)	NS
To be able to share my daily life experiences	High	233 (61.6%)	70 (70.0%)	NS
	Low	29 (7.7%)	4 (4.0%)	NS
To find out how my information is used by researchers	High	231 (61.1%)	82 (82.0%)	*p* < 0.001
	Low	30 (10.1%)	5 (5.0%)	NS
To have access to the names of health care professionals I see and have seen	High	228 (60.3%)	74 (74.0%)	*p* = 0.012
	Low	38 (10.1%)	5 (5.0%)	NS
To be able to connect (with appropriate permissions) with other people with rare bone diseases	High	195 (51.6%)	62 (62.0%)	NS
	Low	48 (12.7%)	8 (8.0%)	NS
To have access to the lists of medicines I am on and medication allergies I have	High	181 (47.9%)	65 (65.0%)	*p* = 0.002
	Low	54 (14.3%)	10 (10.0%)	NS
To be able to share that I have anxiety and self-confidence problems	High	170 (45.%)	52 (52.0%)	NS
	Low	61 (16.1%)	12 (12.0%)	NS
To be able to share my experience in making friends, socialising and having relationships	High	151 (39.9%)	55 (55.0%)	*p* = 0.007
	Low	69 (18.3%)	8 (8.0%)	*p* = 0.01

Denominators for percentages are from completed questions and therefore varied between questions. Results show n (%) for high interest scoring 8 to 10 out of 10 and low interest for scores 1 -3 out of 10. Chi-squared significance shown. Fischer Exact test score used if cell count is less than 10 NS = *p* > 0.05

Only two preferences for database features differed between respondents with OI vs respondents with other rare bone and mineral conditions. Those with OI were more likely to highly rate (a) “To allow drug companies across the world access to my unidentified information (anonymised) for research approved by the Rare Bone Diseases European Reference Network, BOND” (70.0 vs 61.2% *p* = 0.04); and (b) “To help educate and increase the knowledge of doctors and health care professionals” (94.5 vs 89.3%, *p* = 0.04); and less likely to highly rate “To help find ways to get an earlier diagnosis” (75.2 vs 83.5% *p* = 0.03), although these differences were small.

**Fig. 2:**
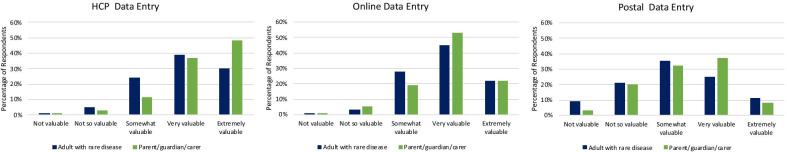
How valuable respondents graded different methods of entering data into the research database. Proportion of adults and parents/guardians/ carers (PGC) shown for online data entry, postal and data entry by Healthcare professional (HCP)

Respondents were asked about *how they would like to participate* with the registry. Overall, more respondents ‘very’ or ‘extremely’ valued HCP data entry (72.7%) compared with online (69.1%) and postal (37.7%) data entry (*p* < 0.001) (Fig. 2). Postal contact was not valued by over 30% of adults and 23% of PGCs. When comparing between groups, PGCs were more likely to value HCP completion than adults (*p* = 0.003).

**Fig. 3:**
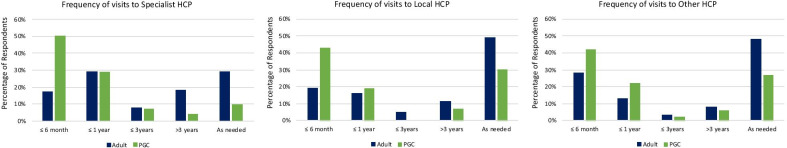
Frequency of specialist, local and other HCP visits

Respondents were asked *how often they saw their specialist* rare bone and mineral condition centre, their local non-specialist HCP or other HCPs (Fig. 3). Other HCPs included physiotherapists, dentists, audiologists as well as other types of doctor (e.g. general practitioner, cardiologist, diabetologist, ophthalmologist, geneticists, endocrinologist, nephrologist, orthopaedist). When comparing groups, 46% of adults and 79% of PGCs reported seeing their specialist HCP at least annually. In individuals with a diagnosis of OI, 30.5% of adults and 68.4% of PGCs reported seeing their specialist HCP at least annually.

Two hundred-five participants submitted additional topics that could be considered for the research database and gave other feedback with overlapping areas raised (Table 4). For additional major topics (Q.34), a total of 76 quotes have been identified and tree categories emerged from the text analysis: (i) quality of life, (ii) healthcare, and (iii) data collection. Regarding to comments on further suggestions (Q.35), a total of 144 quotes were captured and five categories were definied from the text analysis: (i) quality of life, (ii) data collection, (iii) publications, (iv) other, and (v) no further suggestions. There were not statistically significant differences between adults and PGCs. A fuller description of the codes and quote is given in Additional file 3: Table S1 and Additional file 4: Table S2. A summary for patients is available in the Supplmentary materials.

**Table 4:** Participants providing free-text comments

Participants	Q.34^a^ (N = 72)		Q.35^b^ (N = 133)	
	N	%	N	%
Child	0	0.0%	8	6.0%
Adult	53	73.6%	101	75.9%
PGC	19	26.4%	24	18.1%

^a^Q.34: “Is there any major topic which has not been covered in this questionnaire?”; ^b^Q.35: “Is there anything else that you would like to share with us?”

## Discussion

This survey was deployed across 9 EU languages and had a large number of respondents across a range of rare bone and mineral conditions and from adults and PGCs. We observed significant variability by and within each respondent group for content, functionality and data entry options for the research database. A number of additional fields that the stakeholder panel did not propose were also identified across themes related to specific disease features, aspects of care delivery, database functionality and security. Respondents displayed a strong interest to participate in the development of the database, the proposed content items and in keeping updated about registry results. These data will be critical for informing the development of an EU wide research database.

The survey results will expand general features of a rare bone diseases’ impact on people’s lives. Together with improving the databases’ functionality to match the needs of patients, this evidence is hoped to improve recruitment and long-term engagement. A number of classification systems exist for different types of rare disease registries [13–15]. However, the name of the registry is also important for potential participants as it would influence their initial perception and expectations as well as recruitment and engagement. When asked about the preferred and most descriptive name for the registry, research database was the most popular.

All of the proposed database features were valued as interesting by most respondents. There were few differences between adults and PGCs. Some features were consistently valued lower than others, in particular those related to mental health and socialisation. The patient representatives in the authoring group observed that many adults living with rare diseases with significant functional limitation can be reluctant to discuss negative issues around mental health and wellbeing with others. This has not been reported in the literature before and is compounded by the absence of mental wellbeing assessment in some best practice guidelines for clinicians [16]. Assessing mental health in adults with rare bone diseases is likely to be important as many adults with rare bone and mineral conditions have function limitations and increased pain [17] and a number of studies have demonstrated these characteristics are important predictors of mental health and suicide risk in the general population across all ages [18–22]. However, few studies have reported the rate and determinants of suicide in people with rare bone and mineral conditions [23–27]. So, while the survey respondents did not prioritise assessment of aspects of their mental wellbeing, to omit this from the registry was considered counterproductive. More work is needed to understand why these specific items were deprioritised, identify the types of tools to use and how to use them sensitively in the research database to maintain participant engagement, especially as those with mental health problems may be less likely to engage with research database.

*Additional features* were also recommended. These included disease specific topics such as other body systems (hearing, dentistry, pain, sleep, motherhood and sexual health), impacts on specific aspects of living (financial, home care, mobility) as well as the wider family unit to including partners/ spouse/ children.

Another theme was around *healthcare provision* including access to doctors, medicine and allied health professionals, complaints and coordination. A number of responses raised the issue of trust in the HCPs as part of the medical-partient relationship. Trust is established as a fundamental component of a healthy relationship between patient and HCP [28]. Low levels of trust by patients predicts poorer patient outcomes in common [29, 30] and rare [31] diseases. However, there is a paucity of evidence for levels of patient reported trust with their specialist and non-specialist HCPs in the rare disease setting, where lack of specialist knowledge may be an important factor. We therefore recommend tools to measure HCP trust are included in the research database specification.

Concerns around *data protection and confidentiality* were also raised. Issues were raised around confidentiality of the collected data, who would have access to the data and potential abuse of the database from cyber crimes. This highlights the need for clear communication of the research governance strategy that includes participants. The survey demonstrated that while sharing of collected data with clinicians, researchers and companies was rated highly by most respondents, it was not unanimous, underlying the importance of offering a preference or dynamic consent model [32] for the research database to be as inclusive and therefore as generalisable as possible. This also potentially blurs the distinction between use of the database for research vs. informing clinical care from the perspective of respondents. Another finding was the need to share the results of the survey. To address this, the patients group representatives have produced a multi-language summary (see Additional file 1) that will be translated and published in the newsletters of the patient groups who participated in addition to the BOND website for feedback of information. A key next step is to identify and deliver a process of evaluating these findings so they can be operationalised as a core generic outcome for an EU rare bone and mineral condition research database as well as other databases.

The cornerstone for any study of natural history is regular and representative information about the patients studied. The usual method for capturing the natural history for rare bone studies is using HCP visits to trigger data entry. While respondents valued as ‘very’ or ‘extremely interested’ in HCP data entry for generating longitudinal data, the findings from the survey have identified potential problems with using this approach. Over half of adults reported seeing their specialist HCP less than once a year. This may lead to gaps in data collection and a potential bias as patients with less severe disease would have more incomplete data, that could limit the genralisability of findings. This is a common issue raised in the field [8, 33, 34]. Another approach could be to use routine healthcare data to collect information about hospital admissions and other healthcare data that is not dependent on patients visiting specialist HCPs. However, there are significant limitations with this approach, primarily around diagnostic certainty. A recent UK based study using routine health data has demonstrated for patients with familial rickets, there is significant misclassification if only routine primary care coding is used [35]. Another limitation is that outcomes that are important to people, such as measures of quality of life, are absent from routine healthcare data. In addition, routine healthcare data differ significantly between different countries that may make comparisons difficult. These findings highlight the need to explore options for registries to collect both information from clinicians and patients [36] to improve data quality [6, 37]. To deliver this requires greater engagement of individuals with rare bone and mineral conditions. Incorporating some of the suggested content from this survey would be a first step to achieving this.

The strengths of this survey are based around its multiple languages and the large response rate. The major limitations include the substantial proportion of respondants who did not complete the preference section of the survey and the lack of clinical confirmation for the rare bone and mineral diagnoses. This may be particularly relevant for those respondents who did not know the name of their rare bone or mineral condition. Also the modest number of children taking part limits the generalisability for findings from children and may account for the unexpectedly lower number of children who preferred online methods of engagement. The low number of children taking part may reflect the lack of representation of children in the stakeholder group. This should be addressed by future surveys. Other major limitations included the lack of data on country of origin, incomplete coverage of all European languages, lower potential recruitment over the summer period and the unknown non-response rate and the high proportion of responses from one diagnostic group (OI). The high number of ineligible patients is another limiting factor. Reasons might be a misunderstandig of the survey’s aims and inclusion criteria. Due to the nature of this survey it was not possible to collect personal and identifiable information of the respondents such as age.

The experience from launching this patient focused survey has led to the following recommendations: 1. The survey is launched across multiple languages at the same time (not piloted in one language) as what exists in social media/web exists for everyone and will be shared; 2. Country of residence and first language is included; 3. Translations are checked manually by multiple natural speakers including representatives from user groups before launch; 4. Patient organizations should be the primary method of dissemination. 5. Including a document to explain in plain language why answering the survey is important and what the results will be used for; 6. A clear deadline for how long the survey will be available and timing of reminders; 7. Direct links to all the language versions of the survey from a single webpage; 8 Differentiation of the type of outcomes in terms of patient reported outcomes vs. clinical / laboratory outcomes; 9. Identify more clearly who can participate and the need to complete all parts of the survey (e.g. please proceed to complete this suvery if you have a genetic diagnosis of a rare bone and mineral condition: skeletal dysplasia or metabolic linked bone condition).

In conclusion, this survey, which focused on individuals with rare bone and mineral conditions, their parents, guardians and carers has produced the first overview of the key issues for an EU-based rare bone and mineral condition research database. The survey demonstrated that using only specialist centre visits for data collection, while preferred by patients, will miss a number of individuals. Combined HCP and patient platforms will be required to collect representative and complete natural history data for this patient group.

## Supplementary Information

**Additional file 1:** English version of study.

**Additional file 2:** Interest score distribution by respondant.

**Additional file 3:** Codes from qualitative analysis.

**Additional file 4:** Quotes from qualitative analysis.

## Data Availability

The datasets during and/or analysed during the current study available from the corresponding author on reasonable request.

## Abbreviations

ERN BOND: European Reference Network on Rare Bone Diseases
ePAGs: European Patient Advocacy Group
EU: European Union
HCP: Health Care Provider
OI: Osteogenesis Imperfecta
PGC: Parent, Guardian or Carer
WG: Working Group
