# Supplementary material for: Patients’ priorities and expectations on an EU registry for rare bone and mineral conditions
Source: Orphanet J Rare Dis. 2021 Nov 3;16:463. doi: 10.1186/s13023-021-02069-9 (PMC8564998; doi:10.1186/s13023-021-02069-9)
Supplement: Supplementary file 1 — Additional file 1: English version of study. [file 13023_2021_2069_MOESM1_ESM.docx]

What individuals with rare diseases, their family and carers expect from an EU registry for rare bone and mineral conditions?

Supplementary materials

**Summary for individuals with rare bone and mineral conditions, their parents, guardians and carers**

Who are we?

We are members of the European Reference Network on Rare Bone diseases (ERN BOND) (<http://ernbond.eu/>) and representatives from patient groups for some of the rare bone and mineral conditions in Europe.

Why did we do this?

There is a plan to make one registry for patients with a rare bone and mineral disorder in Europe to measure how these diseases affect people’s lives. We want to understand what adults, children, parents, family and carers would want from the registry

What did we do?

We produced a survey to ask what the registry should be called, what aspects of health and wellbeing should it cover, and how it should be collected. The survey was launched online in 8 European languages.

What did we find?

From 596 responses, “research database” was the preferred name. Other questions respondents wanted to include were how well patients trust their doctors, but did not want to discuss their mental health or difficulties socialising. This may be because people find these topics hard to discuss. You can find other questions in the table below. While respondents preferred their doctor to enter the data, many patients did not see their doctors regularly, so we will probably need a database for doctors linked to one for patients.

What are we going to do next?

The team at ERN BOND will now look at the responses and write down the important questions the research database should contain as well as think about how to ask them in a sensitive and clear way.

What can I do next?

For more information, checking on the BOND website (<http://ernbond.eu/>) for updates on the Natural History and Registries Working Group (WG5) page.

We would like to thank all the patient groups, individuals with rare diseases, their families, guardians and carers without whose support this survey would not have succeeded.
