# Supplementary material for: Patients’ priorities and expectations on an EU registry for rare bone and mineral conditions
Source: Orphanet J Rare Dis. 2021 Nov 3;16:463. doi: 10.1186/s13023-021-02069-9 (PMC8564998; doi:10.1186/s13023-021-02069-9)

## Slide 1
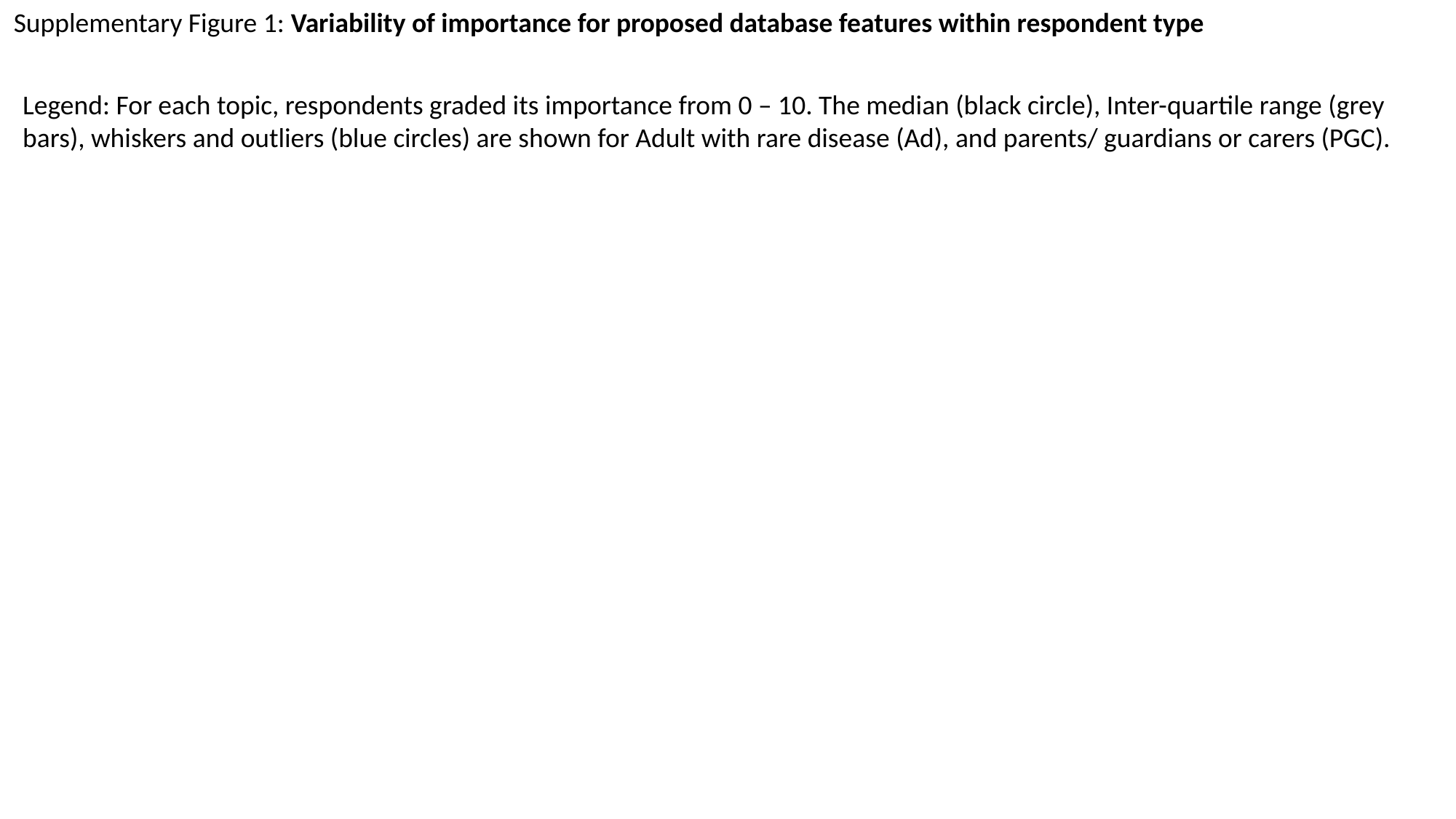

Supplementary Figure 1: Variability of importance for proposed database features within respondent type
Legend: For each topic, respondents graded its importance from 0 – 10. The median (black circle), Inter-quartile range (grey bars), whiskers and outliers (blue circles) are shown for Adult with rare disease (Ad), and parents/ guardians or carers (PGC).

## Slide 2
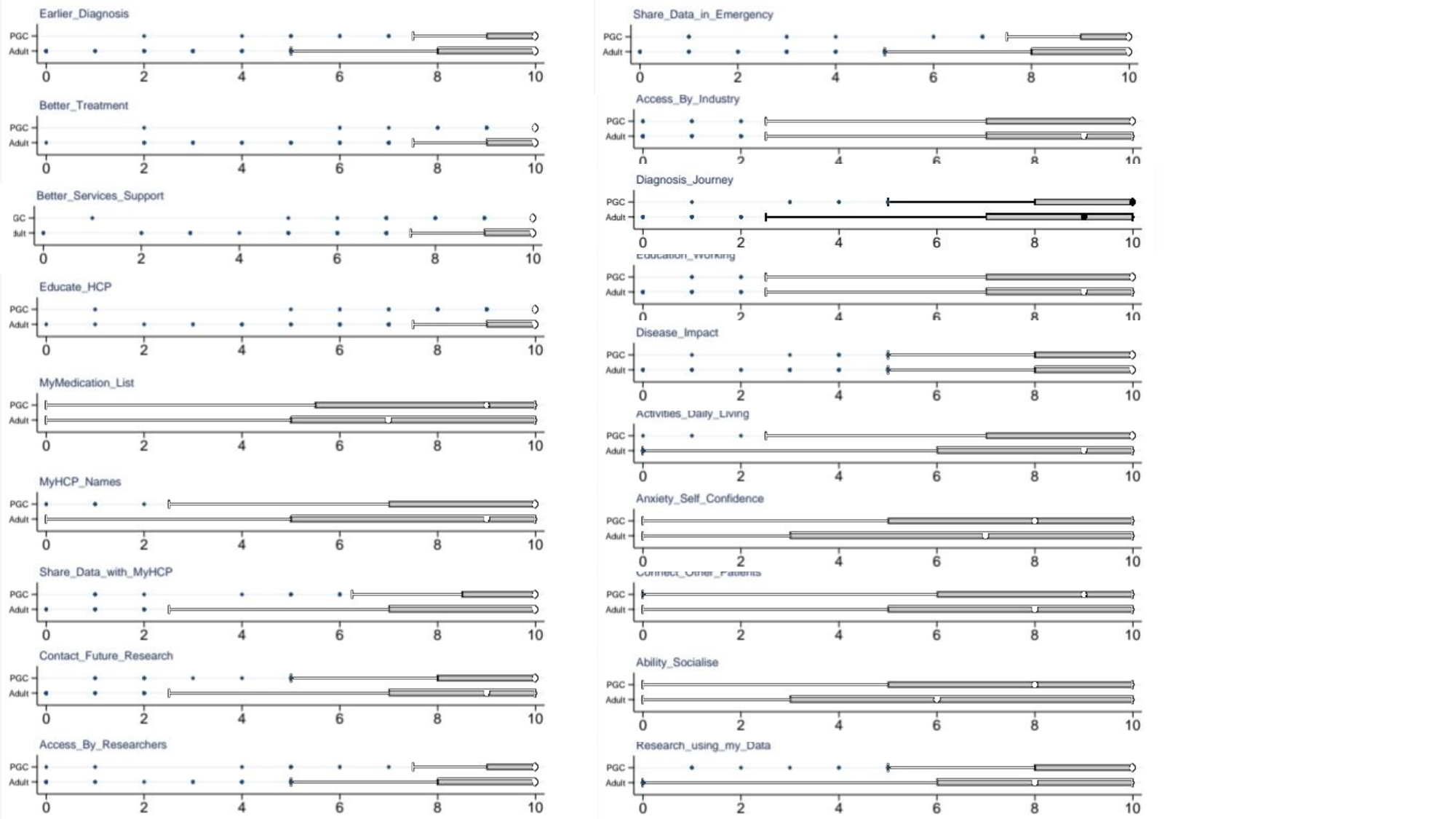

Supplement: Supplementary file 2 — Additional file 2: Interest score distribution by respondant. [file 13023_2021_2069_MOESM2_ESM.pptx]
