# Supplementary material for: Patients’ priorities and expectations on an EU registry for rare bone and mineral conditions
Source: Orphanet J Rare Dis. 2021 Nov 3;16:463. doi: 10.1186/s13023-021-02069-9 (PMC8564998; doi:10.1186/s13023-021-02069-9)
Supplement: Supplementary file 3 — Additional file 3: Codes from qualitative analysis. [file 13023_2021_2069_MOESM3_ESM.docx]

Supplementary Table 1: Categories, codes and quotes from question: Is there any major topic which has not been covered in this questionnaire?

| **CATEGORIES** | **CODES** | **QUOTES** |
| --- | --- | --- |
| Quality of Life | Health-related QoL | *“Sexuality and motherhood in people with rare bone diseases”* (Adult) |
|  | Psychological status | *“the need to be treated as a whole person”* (Adult)  *“the everyday suffering of children”* (PGC) |
|  | Social relationships | *“Effect on partner/spouse/family”* (PGC)  “*Schooling for children”* (PGC) |
|  | Socio-economic status | *“The case of an adult unable to work”* (Adult) |
| Data collection | Type of data | *“[…], disease details and other associated diseases”* (Adult)  *“pathology other than bone”* (PGC)  *“DNA data”* (Adult)  *“[…] ask the patient's age”* (Adult) |
|  | Data management | *“Confidentiality of collected data”* (PGC)  *“[…] It is not clear to me whether he would have access to the data”* (Adult) |
|  | Outcomes | *“What are the main outcomes and measuring points […]”* (Adult) |
| Healthcare | Access to specialistic HCP | *“[…] not all patients get to access specialistic care for rare bone diseases”* (PGC) |
|  | Guidelines/Best practices | *“[…], it would be helpful if there were "best practice" guidelines”* (Adult) |
|  | Medical-patient relationships | *“Trust in medical professionals”* (Adult) |
|  | Treatments | *“Medical follow-up in rare diseases”* (Adult)  *“Transition child-adult”* (PGC)  *“[…] new treatment options”* (Adult) |
