# Supplementary material for: Patients’ priorities and expectations on an EU registry for rare bone and mineral conditions
Source: Orphanet J Rare Dis. 2021 Nov 3;16:463. doi: 10.1186/s13023-021-02069-9 (PMC8564998; doi:10.1186/s13023-021-02069-9)
Supplement: Supplementary file 4 — Additional file 4: Quotes from qualitative analysis. [file 13023_2021_2069_MOESM4_ESM.docx]

Supplementary Table 2: Categories, codes and quotes from question: Is there anything else that you would like to share with us?

| **CATEGORIES** | **CODES** | **QUOTES** |
| --- | --- | --- |
| Quality of Life | Health-related QoL | *“I'm looking for insights on the subject of women or pregnancy”* (*Adult*)  “*How to manage pain related to samaladia”* (Adult) |
|  | Psychological status | *“Yes, the psychological part.... feelings and fears!!!”* (PGC) |
|  | Social relationships | “*Meetings with patients to exchange and play down”* (Adult)  “*Promote encounters only among young people with osteogenesis imperfecta”* (Child) |
|  | Socio-economic status | “*The case of an adult unable to work”* (Adult) |
|  | Patient engagement | *“my experience, my background, our association”* (Adult) |
| Data collection | Data management | **“***I am concerned about the whole idea of a 'registry' in and of itself. This is an easily abused database potentially especially in the age of cyber-crimes”* (Adult)  *“how can I access the data collection/registry/etc […]”* (Child) |
|  | Survey data collection | “*There is a lack of better understanding to be able to better answer”* (PGC)  “*Are you anti-Apple? Can't answer on iPad. It's not easy”* (Adult)  *“Question 29 needs to be rethought”* (Adult) |
| Publications | Guidelines/Best practices | *“There are no physiotherapy best practice treatments for older patients […]”* (Adult) |
|  | Dissemination | *“Information and treatment results”* (Adult) |
|  | Survey’ results | *“I would like to have a feedback about the follow up of this project!”* (Adult)  *“Will the survey results be shared with the participants”* (PGC) |
| Other |  | *“Back to an earlier question, I am not only an adult with a rare disease […]”* (Adult) |
| No additional suggestions | No | *“At the moment that's all”* (Adult) |
|  | Appreciation | *“Thank you for helping us”* (Adult)  *“I appreciate this kind of initiatives very much”* (PGC)  *“yes, thank you for everything you do”* (Adult) |
